# Primary pulmonary lymphoepithelioma-like carcinoma with positive expression of Epstein-Barr virus and PD-L1: A case report

**DOI:** 10.1016/j.ijscr.2021.01.066

**Published:** 2021-01-20

**Authors:** Akihiro Sasaki, Tatsuya Kato, Hideki Ujiie, Yasushi Cho, Masaaki Sato, Mitsuhito Kaji

**Affiliations:** a Department of Thoracic Surgery, Sapporo Minami-sanjo Hospital, Sapporo, Hokkaido, Japan; b Department of Cardiovascular and Thoracic Surgery, Hokkaido University Faculty and School of Medicine, Sapporo, Hokkaido, Japan; c Department of Pathology, NTT East Japan Sapporo Hospital, Sapporo, Hokkaido, Japan

**Keywords:** ALK, anaplastic lymphoma kinase, CT, computed tomography, EBER-ISH, EBV-encoded small ribonucleic acid-*in situ* hybridization, EBV, Epstein-Barr virus, EGFR, epidermal growth factor receptor, ICIs, immune checkpoint inhibitors, IHC, immunohistochemical, LELC, lymphoepithelioma-like carcinoma, NSCLC, non-small cell lung cancer, PD-L1, programmed death-ligand 1, SCC, squamous cell carcinoma, Lymphoepithelioma-like carcinoma (LELC), Epstein-Barr virus (EBV), EBV encoded small RNA (EBER), *In situ* hybridization (ISH), Programmed death-ligand 1 (PD-L1), Case report

## Abstract

**Introduction and importance:**

Pulmonary lymphoepithelioma-like carcinoma (LELC) is a rare type of non-small cell lung cancer (NSCLC) that is classified as a subtype of unclassified carcinoma by the WHO. LELC is usually associated with Epstein-Barr virus (EBV) infection. LELC has often been observed in Southeast Asia; however, it is extremely rare in Japan.

**Case presentation:**

A 60-year-old Japanese woman presented with an abnormal shadow in the left lung on chest radiography. Chest computed tomography showed a nodule located between the lingular and basal anteromedial segments. A blood test suggested an existing EBV infection, and LELC was suspected preoperatively in the transbronchial lung biopsy. She underwent a lingular and basal bi-segmentectomy. The EBV-encoded small ribonucleic acid in-situ hybridization (EBER-ISH) was positive, and she was diagnosed with LELC. Moreover, programmed death-ligand 1 (PD-L1) expression was moderately positive. No recurrence was observed for 30 months.

**Clinical discussion:**

Although LELC has been reported as a low-grade malignancy with a good prognosis, the frequency of PD-L1 expression in LELC seems to be higher than that in other NSCLCs. Moreover, it has been reported that LELC patients with high PD-L1 expression are likely to have early recurrence/metastasis and poor prognosis.

**Conclusion:**

An investigation of PD-L1 expression for LELC would be useful considering the benefit of PD-1/PD-L1 blockade in patients with pulmonary LELC with high PD-L1 expression. The present case is the first report of LELC with positive expression of EBER-ISH and PD-L1 in Japan.

## Highlights

• Pulmonary lymphoepithelioma-like carcinoma (LELC) is extremely rare in Japan.
• None of the studies regarding LELC in Japan described an association with PD-L1.
• This is the first case of LELC in Japan with both expression of EBER-ISH and PD-L1.
• An investigation of PD-L1 would be useful considering the PD-1/PD-L1 blockade.
• Thus, PD-L1 expression should be examined in patients with pulmonary LELC.

## 1 Introduction

Pulmonary lymphoepithelioma-like carcinoma (LELC) is a rare type of non-small cell lung cancer (NSCLC) that is classified as a subtype of unclassified carcinoma by WHO. LELC is usually associated with Epstein-Barr virus (EBV) infection and is more prevalent in Southeast Asia. However, it is extremely rare in Japan as EBV infection is uncommon [1]. Herein, we present a case of a Japanese woman with primary pulmonary LELC, along with a review of the literature. This is the first report of simultaneous EBV and programmed death-ligand 1 (PD-L1) positivity in a patient with LELC in Japan. This manuscript has been reported in line with the SCARE 2020 guidelines [2].

## 2 Presentation of case

**Fig. 1:**
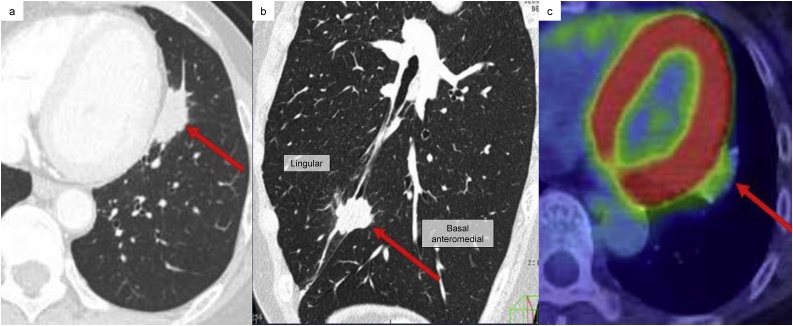
Radiological imaging findings. **a, b**) Computed tomography (CT) shows a nodule located between the lingular and basal anteromedial segments in the incomplete lobulation. (a; Axial, b; Sagittal image). **c**) Positron emission tomography (PET)-CT shows abnormal uptake in the tumour with standardized uptake value max of 2.8. The red arrow indicates the tumour.

A 60-year-old Japanese woman presented with an abnormal shadow in the left lung on chest radiography during a medical examination. The patient was referred to our hospital by walking for further investigation and treatment. The patient had no medical or drug history and no genetic family history. Chest computed tomography (CT) showed a 2.7 cm irregular nodule with spiculation located between the lingular and basal anteromedial segments in the incomplete lobulation. The tumour was adjacent to the pericardium but had no obvious invasion (Fig. 1a and b). ^18^F-fluorodeoxyglucose positron emission tomography showed abnormal uptake in the tumour (Fig. 1c). A transbronchial lung biopsy showed large polygonal cells and cytokeratin-positive epithelial clumps with lymphocyte infiltration and proliferation. In blood tests, EBV early antigen-immunoglobulin G (640 times higher) and Epstein-Barr nuclear antigen antibody (80 times higher) were positive, but EBV early antigen-immunoglobulin M was negative (< 10 times higher). This suggested an existing EBV infection. All serum tumour markers were within the normal range. As primary pulmonary LELC was suspected preoperatively, we explained to the patient the stage and the expected prognosis of LELC and obtained her consent for further treatment. Then, we decided to perform surgery firstly. The tumour was located between the lingular and basal anteromedial segments within incomplete lobulation. Considering the residual lung volume and lymphatic and vascular flow pathways [3], we performed lingular and basal bi-segmentectomy with mediastinal lymph node dissection (ND2a-2). The operator was a specialist in thoracic surgery with over 20 years of experience (one of the co-authors).

**Fig. 2:**
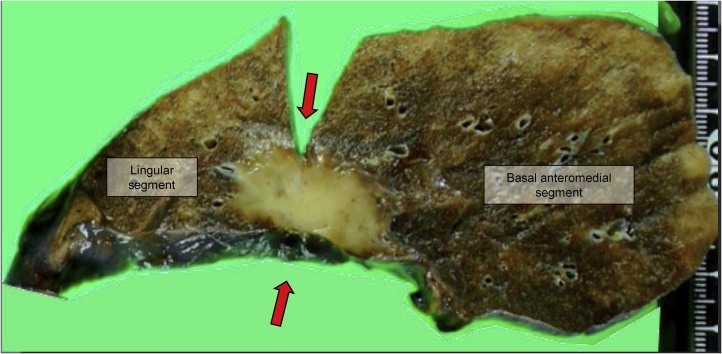
Resected specimen. A tumour is located between the lingular and basal anteromedial segments. Right side: lingular segment, left side: basal anteromedial segment. The red arrows indicate the estimated lobulation line.

**Fig. 3:**
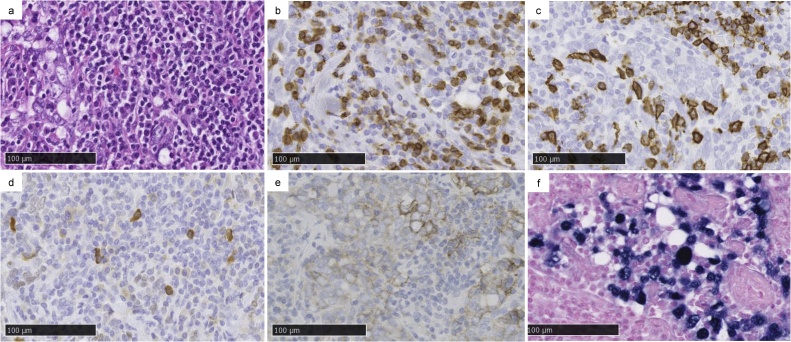
Histopathological and immunohistochemical (IHC) findings and the result of *in situ* hybridization (ISH) for Epstein-Barr virus (EBV)-encoded small ribonucleic acid (EBER). The black bar indicates 100 μm. Prominent lymphocytes and plasma cell infiltration are seen around the interstitium with hematoxylin and eosin staining. **b, c**) Lymphocytes are positive for both CD3 (b) and CD20 (c) in the IHC study. **d**) p40 is slightly positive. **e**) Programmed death-ligand 1 (PD-L1) is moderately positive. **f**) EBER-ISH is strongly positive.

There was a 2.8 cm tumour with pleural indentation in the centre of incomplete lobulation (Fig. 2). Hematoxylin and eosin staining showed atypical cells that proliferated in a nest pattern with the infiltration of CD3 and CD20-positive lymphocytes and plasma cells around the interstitium (Fig. 3a–c). The tumour cells were positive for AE1/AE3 and p40 (Fig. 3d). However, thyroid transcription factor 1 (TTF-1) was negative, suggesting similar characteristics to squamous cell carcinoma (SCC). Genetic mutation analysis revealed that the patient was negative for the epidermal growth factor receptor (EGFR) mutation and anaplastic lymphoma kinase (ALK) rearrangement. PD-L1 expression was moderately positive (%positivity was approximately 1–49%) in the immunohistochemical (IHC) study (Fig. 3e). The nuclei of the tumour cells were positive for EBV-encoded small RNA *in-situ* hybridization (EBER-ISH; Fig. 3f). Therefore, the patient was diagnosed with primary pulmonary LELC. There was no lymph node metastasis or intrapulmonary metastasis (PM0). The tumour was classified as PL3 because it was located in incomplete lobulation. Lymphatic invasion was negative, but vascular invasion was positive, suggesting invasive cancer. The final pathological stage was determined to be p-T2aN0M0, stage IB.

The postoperative course was uneventful. We had judged that the patient could tolerate oral adjuvant chemotherapy because of his relatively young age and good general condition. The patient was informed and consented to receive oral tegafur-uracil and its side effects. The patient has received oral tegafur-uracil for two years as adjuvant therapy. The patient is carefully followed up outpatiently as the tumor was PD-L1 positive suggesting high risk of recurrence. No evidence of recurrence has been observed for 30 months.

## 3 Discussion

This is the first report of LELC with EBER-ISH and PD-L1 expression from Japan. Primary pulmonary LELC is a clinicopathologically distinctive cancer that was first reported in 1987 [4]. It represents 0.9% of all NSCLCs, but is extremely rare in Japan [1,5]. Most LELC cells are positive for CK, CK5/6, AE1/AE3, and p40, similar to poorly differentiated SCC in IHC [6]. The presence of EBV in the tumour cells must be proven to diagnose LELC according to the WHO classification. EBER-ISH is of value in its differential diagnosis. In our case, TTF-1 was negative, and p40 was positive, indicating SCC. Further, nuclei of the tumour cells were positive for EBER-ISH, indicating LELC.

In terms of Japanese cases of pulmonary lymphoepithelioma-like carcinoma, there have been 13 cases, including our case, that underwent surgical resection (Table 1) [1,[7], [8], [9], [10]]. The age of onset was relatively young, with a mean age of 63.7 (39–83) years. Eight out of thirteen were women, and half were non-smokers, which is consistent with a previous report [1]. The mean tumour diameter was 3.3 (1.0–9.0) cm. In all cases, except for our case, lobectomies were performed. In our case because the tumour was located in the centre of incomplete lobulation, bi-segmentectomy was performed considering radical cure and preservation of lung function. This made it possible to remove the tumour with sufficient margins.

**Table 1:** Cases of surgically resected pulmonary lymphoepithelioma-like carcinoma in Japan.

Case	Source	Age/Sex	BI	Location	Size	p-Stage	Operative	Adjuvant therapy	Recurrence site/Therapy	Prognosis
	(year)				(cm)	(TNM)	Procedure			(period)
1	Higashiyama	55/M	1140	RML	2	IA2 (T1bN0M0)	Lobectomy	no	no/-	AW (60 m)
	(1995)									
2	Higashiyama	65/M	1600	RUL	3.5	IB (T2aN0M0)	Lobectomy	no	no/-	AW (60 m)
	(1995)									
3	Muraisi	39/F	0	RML	4	IIIB (T2aN3M0)	Bi-lobectomy	RT, CBDCA + VP-16	no/-	AW (32 m)
	(1999)									
4	Abe	57/M	0	LLL	4.5	IVA (T2bN0M1a)	Lobectomy	no	Pleural/CBDCA + PTX	AWD (60 m)
	(2004)									
5	Kobayashi	67/F	n.a	RML	3	IIB (T1cN1M0)	Bi-lobectomy	RT	no/-	AW (60 m)
	(2004)									
6	Matsutani	73/M	800	LLL	2.5	IA3 (T1cN0M0)	Lobectomy	no	Bone/RT, CBDCA + PTX	AWD (39 m)
	(2009)									
7	Iwanaga	65/F	0	RML	9	IIIA (T4N0M0)	Lobectomy	CBDCA + PTX	no/-	AW (5 m)
	(2010)									
8	Imamura	60/F	0	RML	4	IIB (T3N0M0)	Lobectomy	no	Lymph node/RT, CBDCA + PTX	AW (120 m)
	(2012)									
9	Tanaka	71/F	0	LLL	1.7	IIIA (T3N1M0)	Lobectomy	CDDP + VNR	no/-	AW (6 m)
	(2012)									
10	Yanaginuma	83/F	0	RUL	1.9	IIB (T1bN1M0)	Lobectomy	no	no/-	AW (29 m)
	(2013)									
11	Iwabuchi	71/M	1000	RLL	1.3	IA2 (T1bN0M0)	Lobectomy	no	no/-	AW (7 m)
	(2016)									
12	Atsumi	62/F	0	RML	2.5	IA3 (T1cN0M0)	Lobectomy	no	no/-	AW (18 m)
	(2016)									
13	Present case	60/F	0	LUL	2.7	IB (T2aN0M0)	Bi-segmentectomy	UFT	no/-	AW (30 m)
	(2020)									

AW, alive and well; AWD, alive with disease; BI, Brinkman index; CBDCA, carboplatin; CDDP, cisplatin; F, female; LLL, left lower lobe; LUL, left upper lobe; M, male; m, month; -, not applicable; n.a, not available; PTX, paclitaxel; RLL, right lower lobe; RML, right middle lobe; RT, radiotherapy; RUL, right upper lobe; UFT, tegafur/uracil; VNR, vinorelbine detartrate; VP-16, etoposide.

In most reports of pulmonary LELC, the cases were early stage and resectable [11]. Previously, LELC was reported as a low-grade malignancy with a better prognosis than other NSCLCs [11,12]. There was no difference in prognosis compared with stage I NSCLCs. However, the 5-year survival rates of stage II and III/IV have been reported as 62.5% and 60.6%, respectively [11]. Of the Japanese cases, one case with stage I primary tumour (Table 1, serial no. 6) presented with postoperative bone metastases. However, the other cases had a relatively good prognosis, and the 5-year overall and disease-free survival rates were 100% and 67%, respectively. In three patients who received adjuvant chemotherapy with platinum doublet, no recurrence or metastasis was observed regardless of the pathological stage. In our case, although no lymphatic metastasis was observed, vascular invasion was present. Therefore, tegafur-uracil was used as an adjuvant therapy. Currently, the patient has not experienced recurrence; however, careful follow-up is required.

Although LELC is a rare disease, the standard treatment is surgical resection, without consensus regarding adjuvant chemotherapy. No driver gene mutation characteristics of LELC, such as EGFR mutation and ALK rearrangement, have been found [13]. Both EGFR and ALK were negative in our case. The frequency of PD-L1 expression in LELC seems to be higher (91%) than that in other NSCLCs (49.3%). However, it has been reported that LELC patients with high PD-L1 expression are likely to have early recurrence/metastasis and poor prognosis [14]. Importantly, PD-L1 expression is elevated in most EBV-related cancers such as EBV-positive lymphoma, gastric cancer, and nasopharyngeal carcinoma [15,16]. The evidence of such an intimate relationship between LELC and EBV further incriminates EBV as a major player in tumorigenesis. There is some evidence that immune checkpoint inhibitors (ICIs) are effective in EBV-related cancers [15,16]. Altogether, ICIs may be a good option for the treatment of advanced LELC. In fact, there is a case report in which nivolumab was found to be efficacious in cases with advanced LELC and high PD-L1 expression [17]. The mechanism of immune checkpoint blockade in pulmonary LELC remains unclear, but the efficacy of ICIs has been observed in other virus-associated cancers. Therefore, further evaluation of ICIs in patients with pulmonary LELC is warranted.

## 4 Conclusion

Surgically resected LELC is rare in Japan, and none of the studies described an association with PD-L1. We reported the first case of pulmonary LELC in Japan with simultaneous positive expression of EBER-ISH and PD-L1. An investigation of PD-L1 expression in LELC would be useful considering the benefit of PD-1/PD-L1 blockade in a patient with pulmonary LELC with high PD-L1 expression. PD-L1 expression should be examined in this disease.

## Declaration of Competing Interest

No conflict of interest.

## Funding

No funding sources.

## Ethical approval

This case report was exempted from ethical approval from the institution.

## Consent

Written informed consent was obtained from the patient for publication of this case report and accompanying images. A copy of the written consent is available for review by the Editor-in-Chief of this journal on request.

## Author contribution

A.S wrote this manuscript and prepared the manuscript under the supervision of T.K, H.U and M.K.

Guarantor is T.K.

M.K and Y.C had medical conference to decide this surgical procedure and performed surgery.

M.S processed the pathological specimens of the surgical specimens.

All authors read and approved the final manuscript.

## Registration of research studies

Not applicable.

## Guarantor

Tatsuya Kato is the Guarantor for this work.

## Provenance and peer review

Not commissioned, externally peer-reviewed.
